# Cervico-Vaginal Inflammatory Cytokine and Chemokine Responses to Two Different SIV Immunogens

**DOI:** 10.3389/fimmu.2020.01935

**Published:** 2020-08-25

**Authors:** Nikki P. L. Toledo, Hongzhao Li, Robert W. Omange, Tamara G. Dacoba, Jose Crecente-Campo, Dane Schalk, Mohammad A. Kashem, Eva Rakasz, Nancy Schultz-Darken, Qingsheng Li, James B. Whitney, Maria J. Alonso, Francis A. Plummer, Ma Luo

**Affiliations:** ^1^Department of Medical Microbiology and Infectious Diseases, University of Manitoba, Winnipeg, MB, Canada; ^2^Center for Research in Molecular Medicine and Chronic Diseases (CIMUS), Campus Vida, Universidade de Santiago de Compostela, Santiago de Compostela, Spain; ^3^Scientific Protocol Implementation Unit, Wisconsin National Primate Research Center, Madison, WI, United States; ^4^National Microbiology Laboratory, Public Health Agency of Canada, Winnipeg, MB, Canada; ^5^Nebraska Center for Virology, School of Biological Sciences, University of Nebraska-Lincoln, Lincoln, NE, United States; ^6^Center for Virology and Vaccine Research, Beth Israel Deaconess Medical Center, Harvard Medical School, Boston, MA, United States

**Keywords:** HIV, SIV, vaccine, mucosal inflammation, pro-inflammatory cytokine/chemokine(s), non-human primates

## Abstract

Studies have shown that vaccine vectors and route of immunization can differentially activate different arms of the immune system. However, the effects of different HIV vaccine immunogens on mucosal inflammation have not yet been studied. Because mucosal sites are the primary route of HIV infection, we evaluated the cervico-vaginal inflammatory cytokine and chemokine levels of Mauritian cynomolgus macaques following immunization and boost using two different SIV vaccine immunogens. The PCS vaccine delivers 12 20-amino acid peptides overlapping the 12 protease cleavage sites, and the Gag/Env vaccine delivers the full Gag and full Env proteins of simian immunodeficiency virus. We showed that the PCS vaccine prime and boosts induced short-lived, lower level increases of a few pro-inflammatory/chemotactic cytokines. In the PCS-vaccine group only the levels of MCP-1 were significantly increased above the baseline (*P* = 0.0078, Week 6; *P* = 0.0078, Week 17; *P* = 0.0234; Week 51) following multiple boosts. In contrast, immunizations with the Gag/Env vaccine persistently increased the levels of multiple cytokines/chemokines. In the Gag/Env group, higher than baseline levels were consistently observed for IL-8 (*P* = 0.0078, Week 16; *P* = 0.0078, Week 17; *P* = 0.0156, Week 52), IL-1β (*P* = 0.0234, Week 16; *P* = 0.0156, Week 17; *P* = 0.0156, Week 52), and MIP-1α (*P* = 0.0313, Week 16; *P* = 0.0156, Week 17; *P* = 0.0313, Week 52). Over time, repeated boosts altered the relative levels of these cytokines between the Gag/Env and PCS vaccine group. 18 weeks after final boost with a higher dosage, IP-10 levels (*P* = 0.0313) in the Gag/Env group remained higher than baseline. Thus, the influence of vaccine immunogens on mucosal inflammation needs to be considered when developing and evaluating candidate HIV vaccines.

## Introduction

Inflammation promotes activation and localization of HIV target cells, and elevated cervico-vaginal inflammation has been associated with higher risk of HIV acquisition (1–6). More recently, genital inflammation has also been shown to reduce the efficacy of anti-HIV microbicides (7). Because CD4+ T cells, an important part of the adaptive immune system, are the main targets of HIV, developing an effective HIV vaccine is more challenging than for other infectious pathogens. Vaccines to infectious pathogens are designed to generate long-lasting, antigen-specific immune memory that can rapidly respond upon re-exposure to a specific pathogen. However, activated CD4+ and CD8+ T cells produce many pro-inflammatory cytokines/chemokines that, in turn, may create more targets for HIV infection (8–12). As a result, vaccine-activated T cells may potentially increase HIV target cells and the susceptibility to HIV infection (13). The magnitude and spectrum of immune responses generated by different candidate HIV vaccines have been studied (14–18). Vaccine encoding full-length Gag/Env would elicit a broader immune response with higher magnitude than a vaccine delivering subsets of peptides. Studies have also shown that live viral vaccine vectors induced immune activation, which may enhance susceptibility to HIV-1 infection (19–22). However, the effect of induction of cervico-vaginal mucosal inflammation by parenterally/nasally-delivered SIV/HIV vaccine immunogens has not been reported. Because vaccine immunogen-induced mucosal inflammation may activate and attract HIV target cells, thus having opposite effect on vaccine efficacy, in addition to evaluating the immune responses generated by different vaccine immunogens, evaluating the mucosal inflammatory cytokines induced by different vaccine immunogens would be important. In this study, we evaluated the changes of cervico-vaginal mucosal inflammatory cytokines in response to immunization and boosts with two SIV vaccines delivering two different SIV immunogens in female cynomolgus macaques. One vaccine delivers full length SIV Gag and Env proteins (Gag/Env vaccine) and the other vaccine delivers twelve 20-amino-acid peptides overlapping the 12 SIV protease cleavage sites (the PCS vaccine) (23, 24). We evaluated the inflammatory cytokine/chemokine levels in the cervico-vaginal lavage (CVL) of female MCMs following immunizations with the two vaccines. The immunogens were delivered by vesicular stomatitis viral vector (VSV) and by NANO formulations.

## Materials and Methods

### Study Design

**Figure 1:**
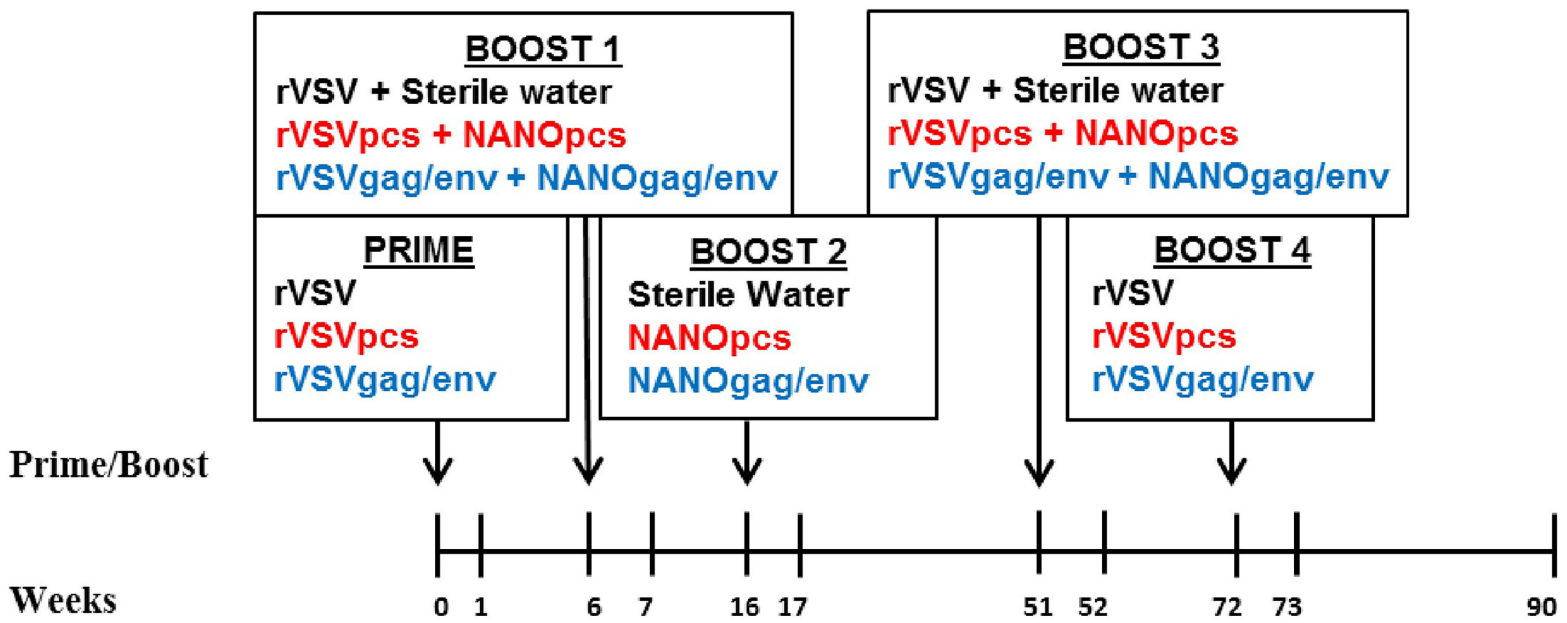
Immunization and sampling scheme. Prime and boosts were given to three groups of 8 female MCMs on the indicated weeks. CVL samples were collected on all time points. Control group received rVSV vector and/or sterile water (black font), PCS group received rVSVpcs and/or NANOpcs (red font), Gag/Env group received rVSVgag/env and/or NANOgag/env (blue font). rVSV, rVSVpcs, and rVSVgag/env were administered intramuscularly. NANOpcs and NANOgag/env boosts were administered intranasally.

Mucosal inflammatory cytokine and chemokine levels in the 24 female MCMs following immunization and repeated boosts were evaluated using a custom 14-plex cytokine/chemokine panel. The monkeys were divided into 3 groups of 8 monkeys that were immunized with their respective vaccines: the control, PCS, and Gag/Env vaccine group. Cervico-vaginal lavage (CVL) from each monkey was collected and analyzed as samples of the mucosal environment following each immunization (Figure 1). CVLs of the 8 monkeys per vaccine group (7 in the Gag/Env group) were run in duplicates and analyzed over the course of 11 time points. Reported protein concentration was normalized against total protein concentration for each monkey at each time point. Levels of each cytokine/chemokine were compared between vaccine groups and between time points within each group.

### Preparation of rVSV Vaccines and NANO Boosts

Generation of rVSVpcs and rVSVgag/env, as well as packaging of the different SIVmac239 immunogens in nanoparticles were performed as previously described (25, 26). Briefly, nucleotide sequences of SIVmac239 (Los Alamos National Laboratory HIV database) encoding 20 amino acids overlapping 12 PCS were previously cloned in pATX VSV-G (rVSV vector), and packaged into rVSVpcs virus. Each PCS peptide was also encapsulated in nanoparticles of chitosan and dextran sulfate, and then pooled together for a single dosage containing 50 μg of each peptide. The freeze-dried nanoparticle formulations were stored at 4°C, and reconstituted in sterile water just before the immunization. SIVmac239 full-length Gag and Env coding sequences were previously prepared and packaged into rVSVgag and rVSVenv viruses (rVSVgag/env), as previously described (25). Separate DNA constructs coding for full-length Gag or Env (pVAX1-Gag and pVAX1-Env) were packaged into separate nanoparticles made of chitosan and tripolyphosphate to form the NANOgag/env formulation.

### Animal Model

Twenty-four colony-bred female Mauritian cynomolgus macaques (*Macaca facicularis*) (MCMs), age 6–7 years old, were used in the study [Bio-culture [Mauritius] Ltd.]. All animal work and sample collection were conducted at Wisconsin National Primate Research Center (WNPRC). Briefly, during the course of the study, the non-human primates within the same experimental group were pair-housed in the standard stainless-steel primate cages with visual and auditory access to other conspecifics. Rooms were maintained at 65–70^0^ F with a 12-h light and 12-h dark cycle, starting at 0600 h. Daily standard NHP chow with fruits or vegetables was provided with foraging enrichment devices and activities provided at least weekly. All animals were monitored for health or welfare issues at least twice daily.

MHC (major histocompatibility complex) typing was performed by the Wisconsin Non-human Primate Research Centre Genetics Services as described previously (25). Those with the same MHC haplotypes were grouped to form several MHC-based subgroups. To ensure the monkeys with different MHC haplotypes are evenly distributed in 3 different experimental groups the monkeys of each MHC-based subgroup were randomly assigned to three experimental groups. Similarly, animals were also screened for infections and constantly monitored for hormone levels, menses schedule, age and weight. Differences in hormone, menses levels and weights were also considered when assigning monkeys to vaccine groups to ensure even and random distribution. Our study comprised of three experimental groups, including the PCS vaccine group, the Gag/Env vaccine group, and the control group.

### Immunization

Immunization was performed as previously described (25). Briefly, these monkeys were divided into 3 groups of 8 monkeys: the PCS vaccine group received rVSVpcs and NANOpcs; the Gag/Env vaccine group received rVSVgag/env and NANOgag/env; and the Control group received VSV virus vector and sterile water boosts (Figure 1). One monkey in the Gag/Env group exhibited signs of severe endometriosis that were unrelated to immunization, leading to early euthanization of the animal. Animals received a final dose of 2 × 10^7^ plaque-forming unit (pfu)/animal for the rVSV immunizations administered intramuscularly via the quadriceps muscle, alternating between left and right for each immunization. Specifically, 1.67 × 10^6^ pfu/rVSVpcs type in the PCS group or 6 × 10^6^ pfu of rVSVgag and 6 × 10^6^ rVSVenv in the Gag/Env group. A higher final dose of 1 × 10^8^ pfu/animal was used for Boost 4. All NANO boosts were administered intranasally in 600 μL liquid (50 μg/PCS peptide for the PCS group or total of 500 μg of plasma DNA encoded Gag and Env for the Gag/Env group). The immunization scheme consisted of prime (rVSV) at week 0 followed by boosts at week 6 (rVSVs + NANOs), week 16 (NANOs), week 51 (rVSVs), and week 72 (rVSVs) (Figure 1).

### Sample Collection

Samples were collected as previously described. Briefly, 2–4 ml of CVL samples were collected weekly by non-traumatically rinsing the vaginal lumen of sedated animals with phosphate buffered saline (PBS) and gently flushing five times using the same syringe. During days of immunization, CVL samples were collected before the vaccination. The CVL samples were then frozen and shipped from Wisconsin to Winnipeg and stored in a −80^0^ C freezer until use.

### 14-Plex Cytokine/Chemokine Assay

Cytokines/chemokines were quantified using a custom 14-plex assay based on a previously published Bio-Plex assay method with some modifications (25, 27). Bio-Plex® Amine Coupling Kit (Bio-Rad, Canada) was used to couple 20 μg of capture antibody for each cytokine/chemokine to 1.25 × 10^7^ Bio-Plex Pro™ fluorescently-dyed magnetic COOH Beads, diluted to a stock concentration of 5 × 10^6^ beads/ml. The standard curves were generated from eight 4-fold serial dilutions of the pooled 14 commercial cytokine/chemokine proteins diluted in 1% BSA (Bovine Serum Albumin) in PBS and corresponding antibody pairs (Table 1). To test for possible cross-reactivity between capture antibodies purchased from different companies, a seven-plex assay was initially developed from same-sourced capture antibodies. Subsequently, the other coupled bead types were added to complete the 14-plex panel. Optimization of the final standard curve range and overall assay consistency after the addition of each bead type were attained by keeping the intra- and inter-assay coefficient of variation (%CV) at <20 and 30%, respectively, and the ratio of observed to expected protein concentration (% recovery) for each cytokine at 70–130%, with no bead counts of <50 per well, as recommended. Specificity of the custom assay was validated using Bio-plex Pro 27-plex human cytokine/chemokine protein standards (Bio-Rad) (Table 1).

**Table 1:** Protein standards and antibodies.

**Name**	**Catalog No**.	**Vendor**
**PROTEIN STANDARDS**		
Recombinant Human CXCL8/IL-8 Protein	208-IL	Novus/R&D System
Recombinant Human CCL5/RANTES Protein	278-RN	Novus/R&D System
Recombinant Human GM-CSF Protein	215-GM	Novus/R&D System
Recombinant Human Interferon Gamma Protein	RIFNG	ThermoFisher Scientific
Recombinant Human IL-1 beta/IL-1F2	201-LB	Novus/R&D System
Recombinant Human IL-6 Protein	206-IL	Novus/R&D System
Recombinant Human IL-10 (aa 19-178) Protein	1064-IL	Novus/R&D System
Recombinant Human IL-17A Protein	317-ILB	Novus/R&D System
Recombinant Human CXCL10/IP-10	266-IP	Novus/R&D System
Recombinant Human CCL2/MCP-1 Protein	279-MC	Novus/R&D System
Recombinant Human CCL3/MIP-1 alpha protein	270-LD	Novus/R&D System
Recombinant Human CCL4/MIP-1 beta Protein	271-BME	Novus/R&D System
Recombinant Human TNF-alpha Protein	210-TA	Novus/R&D System
Recombinant Human IL-1 alpha	200-LA	Novus/R&D System
**CAPTURE ANTIBODIES**		
Human CXCL8/IL-8 Mab	M801	ThermoFisher Scientific
Human CCL5/RANTES PAb	P230E	ThermoFisher Scientific
Rat Anti-Human GM-CSF- Unlabelled	10111-01	SouthernBiotech
Human IFNγ MAb	M700A	ThermoFisher Scientific
Human IL-1beta/ IL-1F2 Antibody	MAB601-500	Novus/R&D System
Human IL-6 Mab	M620	ThermoFisher Scientific
Rat Anti-Human IL-10-Unlabelled	10100-01	SouthernBiotech
Human/Primate IL-17/IL-17A Antibody	MAB317-500	Novus/R&D System
Human IP-10/ CXCL10/CRG-2 Antibody	MAB266-500	Novus/R&D System
Human MCP-1/CCL2/JE Antibody	MAB679-500	Novus/R&D System
Human MIP-1α/CCL3Antibody	AF-270-NA	Novus/R&D System
Human MIP-1β/CCL4 Antibody	MAB271-100	Novus/R&D System
Human TNFα Mab	M303	ThermoFisher Scientific
Human IL-1 alpha/IL-1F1 Mab	MAB-200	Novus/R&D System
**DETECTION ANTIBODIES**		
Human CXCL8/IL-8 MAb, Biotin-labeled	M802B	ThermoFisher Scientific
Human CCL5/RANTES MAb, Biotin-labeled	M230B	ThermoFisher Scientific
Rat Anti-Human GM-CSF-BIOT	10112-08	SouthernBiotech
Human IFNγ MAb, Biotin-labeled	M701B	ThermoFisher Scientific
Human IL-1beta/IL-1F2 Biotinylated Antibody	BAF201	Novus/R&D System
Human IL-6 MAb, Biotin-labeled	M621B	ThermoFisher Scientific
Rat Anti-Human IL-10-BIOT	10110-08	SouthernBiotech
Human/Primate IL-17/IL-17A Biotinylated Antibody	BAF317	Novus/R&D System
Human IP-10/CXCL10/CRG-2 Biotinylated Antibody	BAF266	Novus/R&D System
Human MCP-1/CCL2/JE Biotinylated Antibody	BAF279	Novus/R&D System
Human MIP-1α/CCL3 Biotinylated Antibody	BAF270	Novus/R&D System
Human MIP-1β/CCL4 Biotinylated Antibody	BAF271	Novus/R&D System
Human TNFα MAb, Biotin-labeled	M302B	ThermoFisher Scientific
Human IL-1 alpha/IL-1F1 Biotin-labeled	BAF-200	Novus/R&D System

### Cytokine and Chemokine Quantification

The final panel consisted of 14 (pro- or anti-) inflammatory cytokines/chemokines including Granulocyte Macrophage Colony Stimulating Factor (GM-CSF), Interleukin (IL)-1α, IL-1β, IL-6, IL-8, IL-10, IL-17, Interferon inducible protein 10 (IP-10), Interferon gamma (IFN-γ), Monocyte Chemoattractant Protein-1 (MCP-1), Macrophage Inflammatory Protein-1 alpha/beta (MIP-1α/β), Regulated on activation, normal T cell expressed and secreted (RANTES)/CCL5, and Tumor Necrosis Factor alpha (TNF-α) to quantify the inflammatory response in the CVL samples (Table 1). Cytokines/chemokines that were not detected in ≥ 50% of the samples were excluded from the heat map analysis (TNF-α, IL-1α, and IL-10).

Briefly, 50 μl of capture antibody-coupled magnetic bead complex in 1:600 dilutions (~420,000 beads) were used per well of a 96-well Bio-Plex Pro^TM^ 105 flat bottom plate. Fifty microliter of undiluted CVL sample or standards were analyzed in the assay with assay buffer as general diluent and blank controls. Protein concentrations (pg/ml) were generated and reported by the Bio-Plex Manager 6.1 software (Bio-Rad).

### Total Protein Quantification

Total protein concentration of each sample was used to normalize cytokine/chemokine concentrations to account for possible dilution variations introduced during CVL collection. Total protein concentration (pg/mg)were quantified using NanoOrange® protein quantitation kit (Thermo Fisher Scientific, Waltham, MA) according to manufacturer's protocol, Briefly, standard BSA solution diluted with the NanoOrange working solution was used to generate a standard curve. Fluorescence was measured using a Spectrophotometer Microplate Reader (Spectramax®, San Jose, CA). All samples were run in duplicates with triplicate dilutions.

### Statistical Analysis

Graphing and statistical analysis were performed using GraphPad Prism® 7 statistical software (San Diego, USA). Analyses were done using Wilcoxon matched-pairs signed-rank paired *t*-test and Mann-Whitney *t-*test, for matched pairs and unpaired group comparisons, respectively. Technical duplicates of *n* = 8 samples per group were used for all experiments. Data was not corrected for multiple comparisons. *P-*values lower than 0.05 were considered to be statistically significant.

## Results

### MCMs Primed With rVSV, rVSVpcs, or rVSVgag/env Had Increased CVL Cytokine/Chemokine Levels

**Figure 2:**
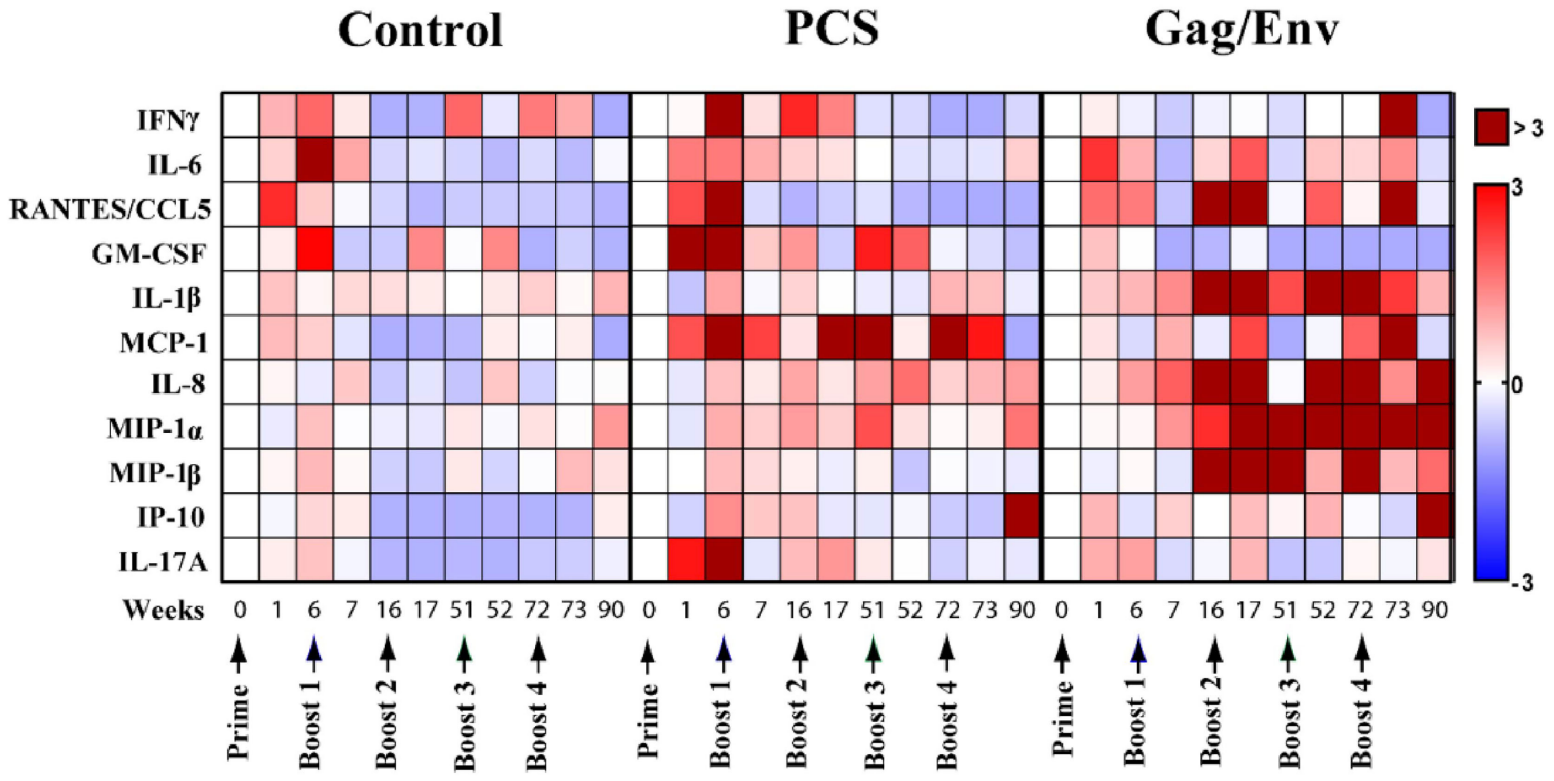
Median cytokine/chemokine fold-change levels at each time point relative to group baseline (Week 0) in all 3 vaccine groups. The fold change was calculated as the ratio of the concentration to the baseline where baseline was set to 0. Arrows indicate each immunization. Bar = Fold change, Shades of blue = downregulated, Shades of red = upregulated. *n* = 8 monkeys/group (*n* = 7 for Gag/Env). Experiments were run in duplicates.

To evaluate the influence of vaccine immunogens on CVL inflammatory mediators, we first assessed the fold change of the cytokine/chemokine levels after prime and each boost (Figure 2). We observed a general fold increase (FI) in the level of majority of cytokines/chemokines in all 3 vaccine groups after prime. Specifically at week 6, a 3-FI was observed in levels of IL-6 (3.19) in the CVLs of MCMs immunized with VSV vector control. A more than 3-FI in IFNγ (4.6), RANTES (4.8), GM-CSF (3.4), MCP-1 (4.4), and IL-17A (3.3) levels were observed in the PCS vaccine group. In the Gag/Env vaccine group, only a 2.4-FI was observed for IL-6, and lower FIs were detected in the level of other inflammatory mediators.

### CVL of MCMs in the Gag/Env Group Had Increased Cytokine/Chemokine Levels Following Multiple Boosting

A general decrease in CVL cytokines/chemokines was observed following multiple boosting with the VSV vector control (Figure 2). Overall, boosting with a combined regimen of rVSVpcs/NANOpcs appeared to have only a modest influence on CVL cytokine/chemokine levels. In comparison with the baseline (week 0), Boost 1 with the PCS vaccine only induced ~2-FI of MCP-1 (2.23), IL-6 (2.27), and IFNγ (2.5). Boost 2 with NANOpcs only resulted in a 4-FI (4.37) in MCP-1 level. Further boosting with rVSVpcs/NANOpcs had little effect on majority of the cytokines/chemokines, except for MCP-1 levels at week 72 (FI: 3.3). One week after a higher dosage of rVSVpcs at Boost 4, majority of the cytokines remained lower than baseline except for a moderate FI in MCP-1 (2.73). By week 90, only the levels of IP-10 (3.05), and MIP-1α (1.62) were higher than week 0.

In contrast, a more sustained and higher-than-baseline increase in cytokine/chemokine levels was observed in the Gag/Env vaccine group following multiple boosts (Figure 2). After boost 1, FI values ranging from 3.3 to 5.9 in RANTES (4.1), IL-1β (5.9), IL-8 (5.3), and MIP-1β (3.3) were observed at week 16, while MIP1-α increased 2.5-fold. Following Boost 2 with NANOgag/env, most cytokine/chemokine levels remained higher than baseline. FI values from 4.55 to 12.96 in RANTES (6.64), IL-1β (12.3), IL-8 (4.55), MIP-1α (7.69), and MIP-1β (4.63) were observed at week 17. Furthermore, the elevation of MIP-1α (4.84), and MIP-1β (12.96) were maintained at week 51. A > 8-fold increase in IL-1β (14.33, 14.43), IL-8 (41.67, 4.7), MIP1α (6.68, 10.4), and MIP-1β (8.37) were observed one and/or 21 weeks after Boost 3, with more than 40 FI in IL-8 at week 52. Following a higher dosage of rVSVgag/env boost, levels of several CVL inflammatory cytokines/chemokines remained elevated compared to baseline (RANTES, 3.22; MCP-1, 4.94; MIP1α, 5.11). Specifically, at week 73 an 11-FI in IFNγ (11.33) and close to 5-fold increase in MCP-1 (4.94) levels were observed, which were not evident after earlier immunizations. Furthermore, the levels of IL-8 (3.7), MIP1α (9.67), IP-10 (9.64) remained elevated at Week 90.

### Actual Levels of Multiple CVL Cytokines/Chemokines Remained Elevated Following Immunization and Multiple Boosting in the Gag/Env Vaccine Group Compared to Pre-immunization and Pre-boost Levels

**Figure 3:**
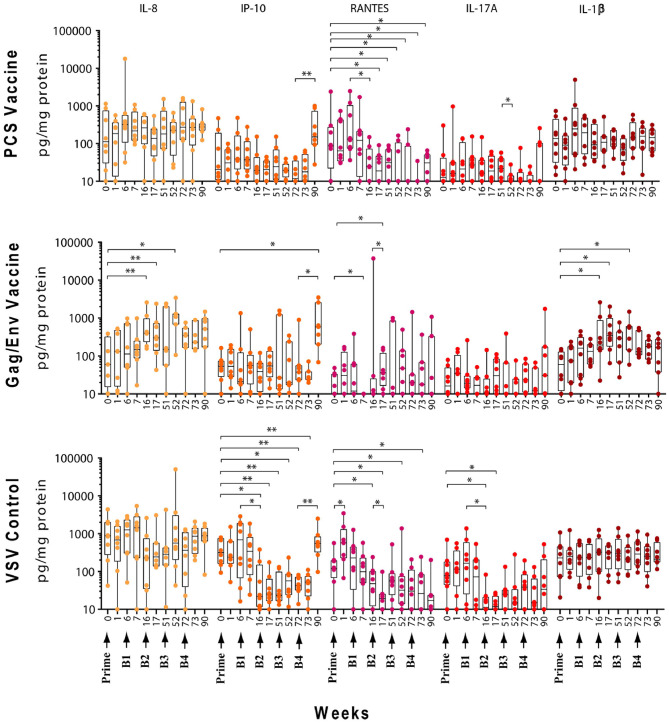
Significant changes in the CVL levels of IL-8, IP-10, RANTES, IL-17A, and IL-1β after immunization and multiple boosts analyzed using Wilcoxon matched- pairs signed-rank paired *t*-test. Data presented as values from individual monkeys with median and range. Arrows indicate immunization. *n* = 8 monkeys/group (*n* = 7 for Gag/Env). Experiments were run in duplicates. *P < 0.05 and **P < 0.01.

**Figure 4:**
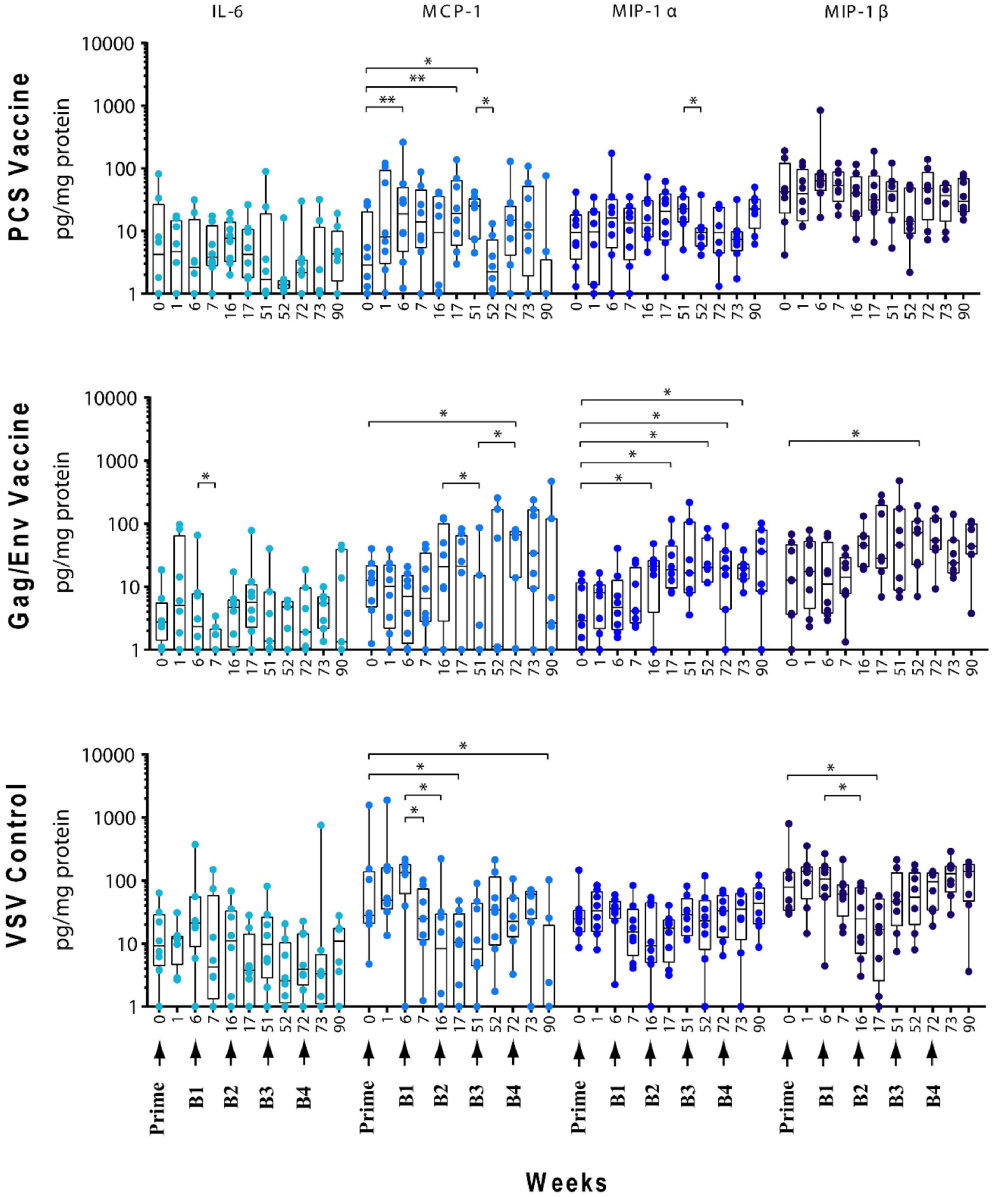
Significant changes in the CVL levels of IL-6, MCP-1, MIP-1α, and MIP-1β after immunization and multiple boosts analyzed using Wilcoxon matched- pairs signed-rank paired *t*-test. Data presented as values from individual monkeys with median and range. Arrows indicate immunization. *n* = 8 monkeys/group (*n* = 7 for Gag/Env). Experiments were run in duplicates. *P < 0.05 and **P < 0.01.

**Figure 5:**
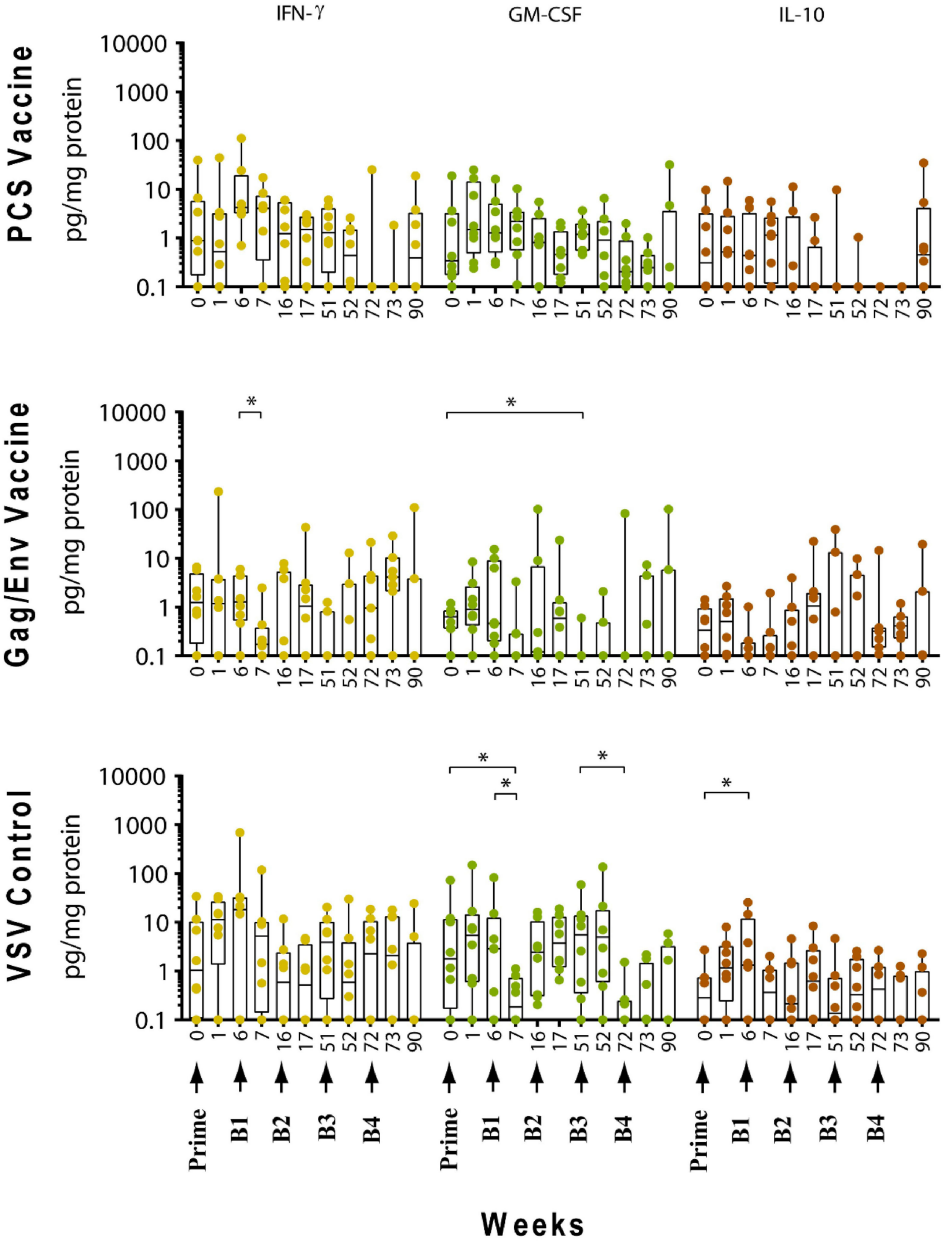
Significant changes in the CVL levels of IFN-γ, GM-CSF, and IL-10 after immunization and multiple boosts analyzed using Wilcoxon matched- pairs signed-rank paired *t*-test. Data presented as values from individual monkeys with median and range. Arrows indicate immunization. *n* = 8 monkeys/group (*n* = 7 for Gag/Env). Experiments were run in duplicates. *P < 0.05.

The levels of CVL cytokines/chemokines at the weeks following prime and each boost were compared using Wilcoxon Paired Matched *t*-test against the pre-immunization level and the level at each boost (Figures 3–5). Significant increase in levels of RANTES (*P* = 0.0156, week 1) and IL-10 (*P* = 0.0156, week 6) were observed when cytokine/chemokine levels were compared before and after prime with the VSV control vector (Figures 3–5). A significant increase in levels of MCP-1 (*P* = 0.0078, week 6) (Figure 4), was observed following prime with rVSVpcs while no significant changes in cytokine/chemokine levels were observed following prime with rVSVgag/env.

#### Boosting With rVSV Vector and Water

Following boosting with the VSV control vector and water (Boost 1), significant decreases were observed in the levels of MCP-1 (*P* = 0.0156, week 7; *P* = 0.0391, week 16), GM-CSF (*P* = 0.0469), IP-10 (*P* = 0.0391), MIP-1β (*P* = 0.0234), and IL-17A (*P* = 0.0156) compared with their pre-Boost 1 levels at week 6 (Figures 3–5). Transient non-significant increases in the levels of some cytokines were also noted. This decreased level of several cytokines following Boost 1 in the control group was also evident in comparison with their pre-immunization levels. The majority of cytokine/chemokine levels following Boost 1 were significantly lower than the pre-immunization levels. Specifically, GM-CSF (*P* = 0.0313, week 7), RANTES (*P* = 0.0391, week 16), IP-10 (*P* = 0.0391, week 16), and IL-17A (*P* = 0.0234, week 16). As expected, no changes were observed in the cytokine/chemokine levels of the control group following sterile water boost (Boost 2), except for the decrease in RANTES levels at week 17 (*P* = 0.0234) in comparison with pre-Boost 2 levels at Week 16 (Figure 3).

In general, lower than baseline levels of RANTES (*P*=0.0156), MCP-1 (*P* = 0.0391), MIP-1β (*P* = 0.0391), and IL-17A (*P* = 0.0156) were observed after Boost 3 in the control group at week 17 while IP-10 levels remained lower until week 51 (*P* = 0.0078). No further significant changes were observed after additional boosting with the control VSV vector except for the significant decrease of GM-CSF (*P* = 0.0469) at week 72 (Figure 5). Although the levels of IP-10 (*P* = 0.0234, week 52; *P* = 0.0078, week 72) and RANTES (*P* = 0.0313, week 52) remained lower than at baseline (Figure 3). Higher dose of VSV vector seemed to have little influence on cytokine/chemokine levels. The only significant increase was observed in IP-10 levels (*P* = 0.0078) at week 90 in comparison to pre-Boost 4 levels at week 72 (Figure 3). However, the elevated level of IP-10 at week 90 was not significantly higher than its baseline levels, while RANTES (*P* = 0.0156) and IP-10 (*P* = 0.0078) at week 73 and MCP-1 levels (*P* = 0.0078) at week 90, were significantly lower than week 0 (Figure 3).

#### Boosting With the PCS Vaccine

Boost 1 (rVSVpcs + NANOpcs) appeared to only modestly influence cytokine/chemokine levels of the PCS vaccine group. Transient and non-significant increases in some cytokines/chemokines were observed, while a significant decrease was observed in RANTES levels (*P* = 0.0156) at week 16 compared to pre-boost levels at week 6 (Figure 3). Interestingly, similar to the control group, RANTES levels remained lower than baseline at week 17 (*P* = 0.0391) and week 51 (*P* = 0.0234) (Figure 3). No significant changes were observed following the NANOpcs boost with the exception of significantly higher than baseline MCP-1 level (*P* = 0.0078) at both week 17 and 51 (*P* = 0.0234) (Figure 4).

Further boosting with rVSVpcs + NANOpcs appeared to have little effect on majority of the cytokines/chemokines. Compared with pre-Boost 3 levels, significant decrease in levels of IL-17A (*P* = 0.0391), MCP-1 (*P* = 0.0234), and MIP-1α (*P* = 0.0156) were observed at week 52 (Figures 3, 4). RANTES level remained lower than that of the baseline (*P* = 0.0391) at week 52 (Figure 3). Transient and non-significant increase in some cytokines/chemokines and a significant increase in IP-10 (*P* = 0.0078) levels at week 90 were observed following boosting with a higher dose of rVSVpcs (Boost 4). However, IP-10 level at week 90 in the PCS vaccine group was not significantly higher than that of pre-immunization baseline (Figure 3). The majority of cytokine/chemokine levels remained lower than baseline, significantly lower level of RANTES was observed at week 72 (*P* = 0.0078), week 73 (*P* = 0.0391), and week 90 (*P* = 0.0391) (Figure 3).

#### Boosting With the Gag/Env Vaccine

In contrast, boosting with rVSVgag/env + NANOgag/env (Boost 1) resulted in a general increase in the CVL cytokine/chemokine levels except for the decrease in IL-6 (*P* = 0.0391) and IFNγ (*P* = 0.0156) levels at week 7. IL-1β (*P* = 0.0234), IL-8 (*P* = 0.0078), and MIP-1α (*P* = 0.0313) levels were significantly higher than the pre-immunization baseline (Figures 3, 4). However, their levels at week 7 were not statistically significantly higher than their levels at week 6 (pre-Boost 1). Significant increase in RANTES levels (*P* = 0.0313) were observed 1 week after the NANOgag/env boost (Boost 2) (Figure 3) while MCP-1 levels (*P* = 0.0313) decreased at week 51 (Figure 4). Following NANOgag/env boost, the levels of several cytokine/chemokines remained significantly higher than baseline, including RANTES (*P* = 0.0391), MIP-1α (*P* = 0.0156), IL-8 (*P* = 0.0078), and IL-1β (*P* = 0.0156) (Figures 3, 4). Only GM-CSF level was decreased and was lower (*P* = 0.0156) than baseline at week 51 (Figure 5).

Additional boost with rVSVgag/env + NANOgag/env (Boost 3) resulted in a general increase in most cytokine/chemokine levels, although only the increase in MCP-1 levels were significant at week 72 (*P* = 0.0469) in comparison with its pre-Boost 3 levels (week 51). Nevertheless, significantly higher than baseline levels were maintained for MIP-1β (*P* = 0.0156), IL-1β (*P* = 0.0156), IL-8 (*P* = 0.0156), and MIP-1α (*P* = 0.0156) at week 52, and for MCP-1 (*P* = 0.0313) and MIP-1α (*P* = 0.0313) at week 72 (Figures 3, 4). Following a higher dose of rVSVgag/env (Boost 4) a general increase in most of the CVL cytokines and chemokines were observed, particularly a significant increase in IP-10 levels (*P* = 0.0156) at week 90 compared to its pre-Boost 4 levels (week 72) (Figure 3). Furthermore, significantly higher than baseline levels of MIP-1α (*P* = 0.0313, week 73) and IP-10 (*P* = 0.0313; week 90) (Figures 3, 4) were observed.

Our study showed that immunization and boosts with different vaccine immunogens influenced inflammatory cytokines/chemokines in the cervico-vaginal mucosa. Higher and more sustained increase in the levels of a broader spectrum of inflammatory mediators was observed following repeated exposure to the Gag/Env immunogens. This elevation of multiple pro-inflammatory mediators in the cervico-vaginal mucosa persisted 18 weeks after the final boost. In contrast, with a more modest and transient increases in CVL inflammatory cytokines/chemokines were evident following repeated exposure to the PCS immunogen.

### Relative Differences in CVL Cytokine/Chemokine Levels Among the Vaccine Groups Were Altered Following Multiple Boosts

**Figure 6:**
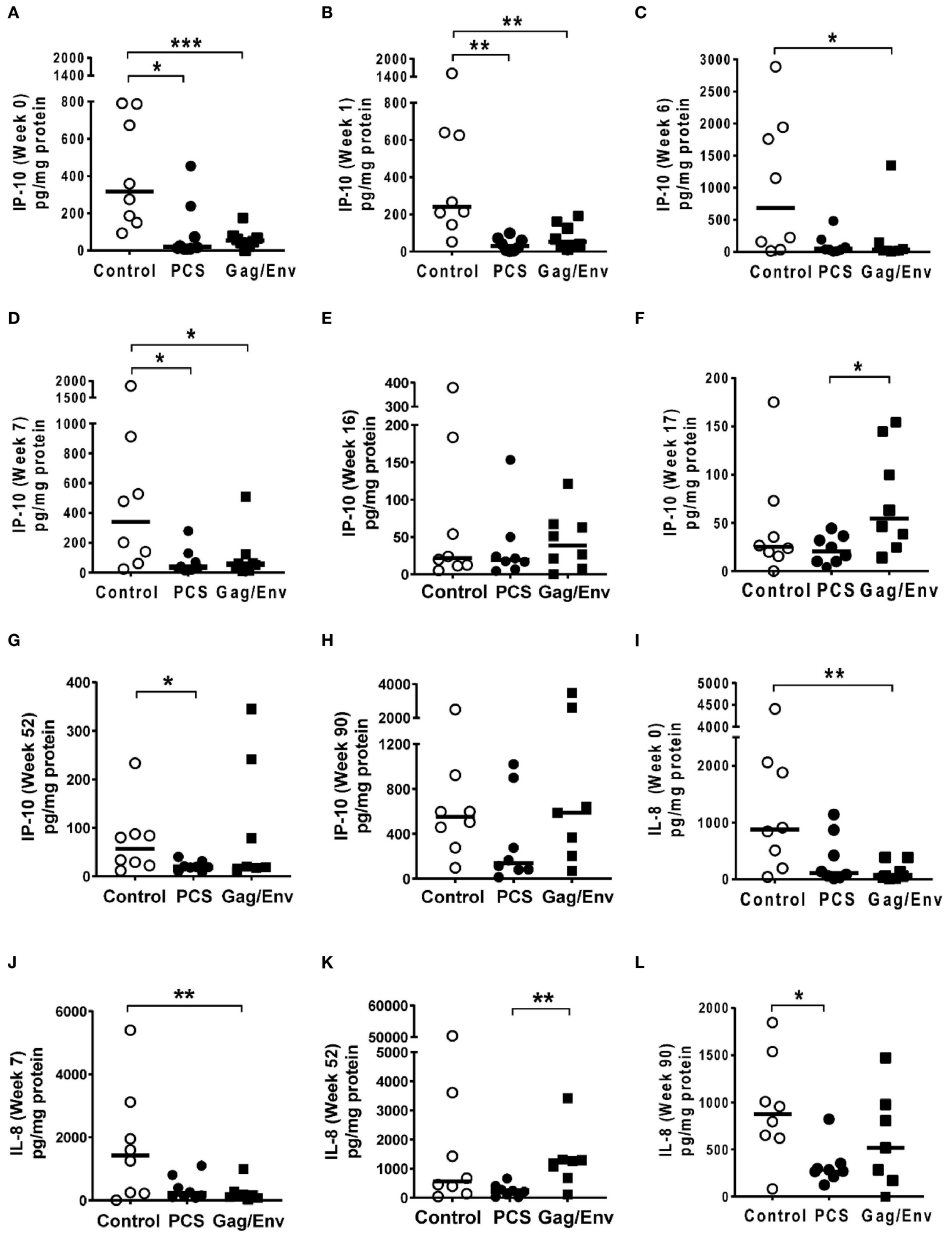
Significant differences in CVL levels of IP-10 and IL-8 among the 3 vaccine groups at different time points including Week 0 and 90 analyzed using Mann- Whitney *t*-test. Data presented as values from individual monkeys with median. **(A–F)** IP-10 **(G–L)** IL-8. *n* = 8 monkeys/group (*n* = 7 for Gag/Env). Experiments were run in duplicates. *P < 0.05, **P < 0.01, and ***P < 0.001. Non-significant differences between groups were not included.

**Figure 7:**
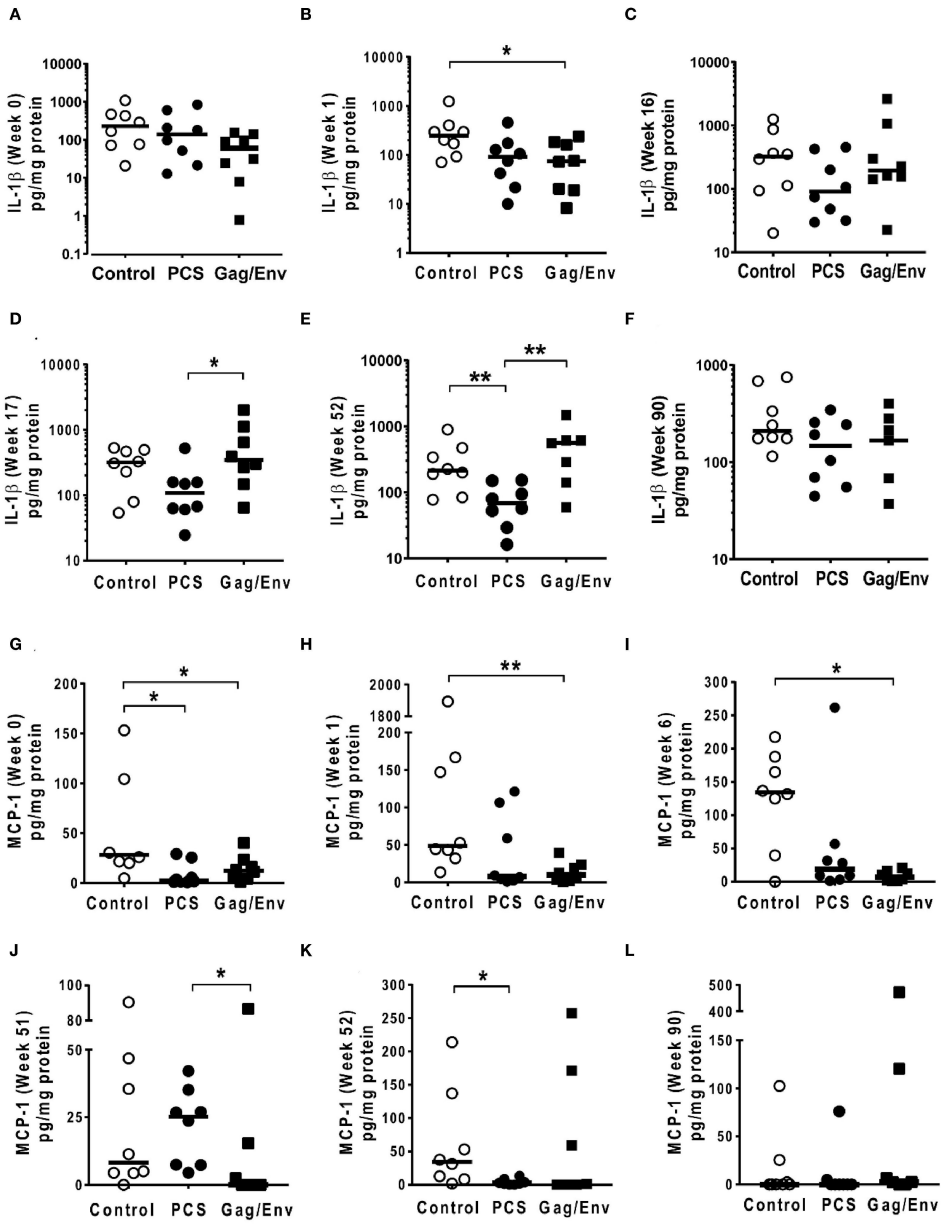
Significant differences in CVL levels of IL-1β and MCP-1 among the 3 vaccine groups at different time points including Week 0 and 90 analyzed using Mann-Whitney *t*-test. Data presented as values from individual monkeys with median. **(A–F)** IL-1β **(G–L)** MCP-1. *n* = 8 monkeys/group (*n* = 7 for Gag/Env). Experiments were run in duplicates. *P < 0.05 and **P < 0.01. Non-significant differences between groups were not included.

**Figure 8:**
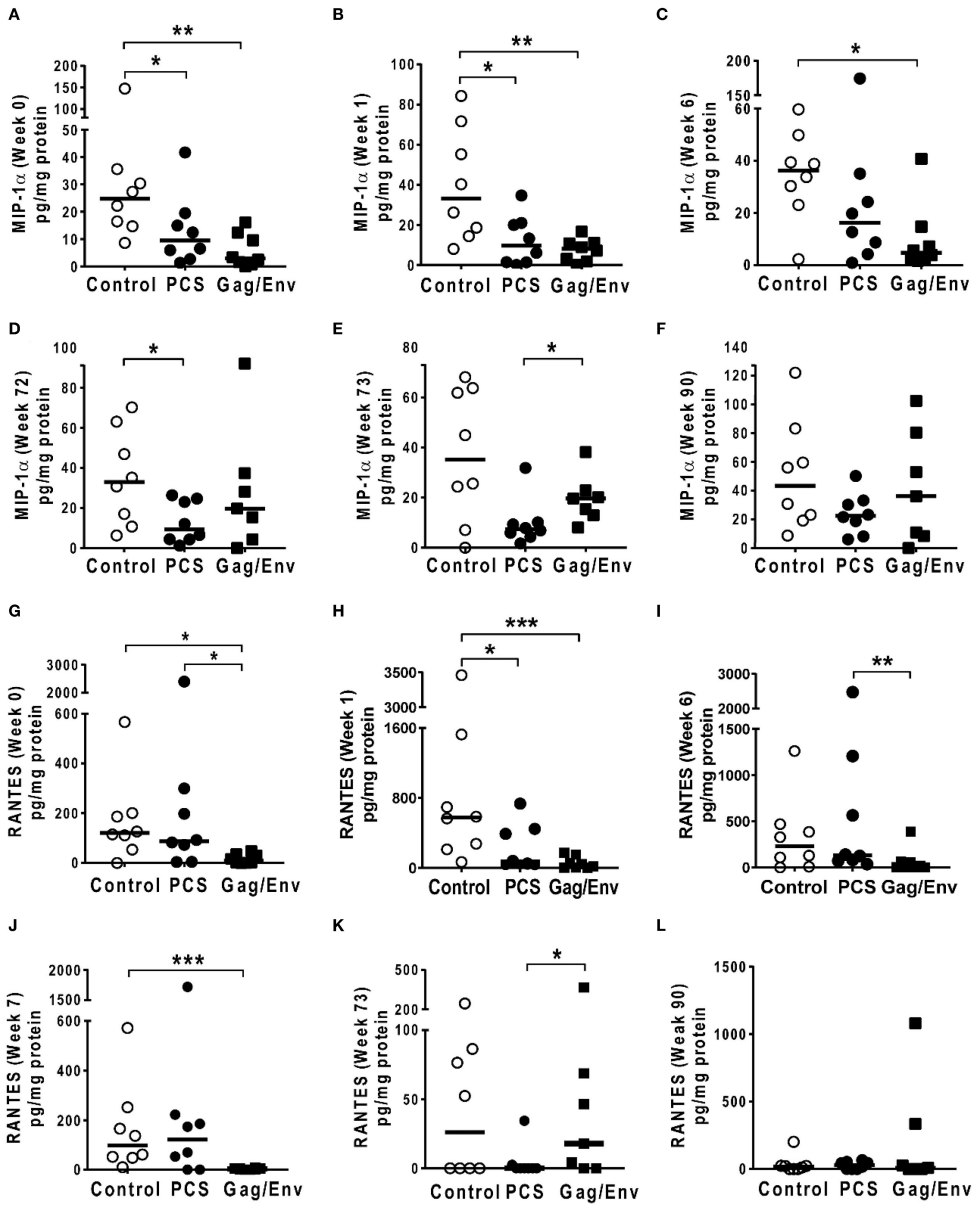
Significant differences in CVL levels of MIP-1α, and RANTES among the 3 vaccine groups at different time points including Week 0 and 90 analyzed using Mann-Whitney *t*-test. Data presented as values from individual monkeys with median. **(A–F)** MIP-1α **(G–L)** RANTES. *n* = 8 monkeys/group (*n* = 7 for Gag/Env). Experiments were run in duplicates. *P < 0.05, **P < 0.01, and ***P < 0.001. Non-significant differences between groups were not included.

**Figure 9:**
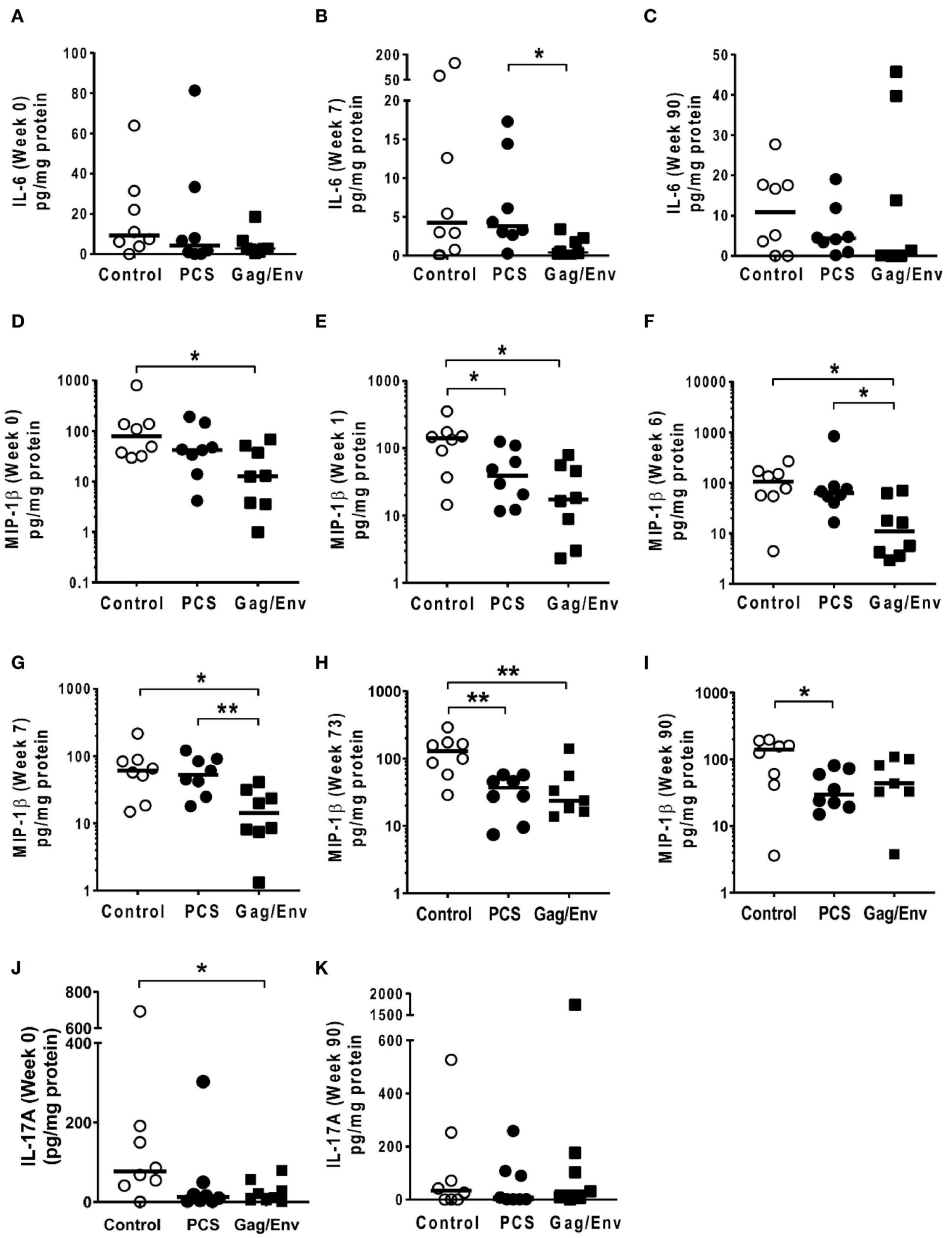
Significant differences in CVL levels of IL-6, MIP-1β, and IL-17A among the 3 vaccine groups at different time points including Week 0 and 90 analyzed using Mann-Whitney *t*-test. Data presented as values from individual monkeys with median. **(A–C)** IL-6 **(D–I)** MIP-1β **(J,K)** IL-17A. *n* = 8 monkeys/group (*n* = 7 for Gag/Env). Experiments were run in duplicates. *P < 0.05 and **P < 0.01. Non-significant differences between groups were not included.

At pre-immunization baseline (week 0), control group CVL levels of IP-10 (*P* = 0.0104, and *P* = 0.0006), IL-8 (*P* = 0.007), MCP-1 (*P* = 0.0205), MIP-1α (*P* = 0.0499, and *P* = 0.0019), RANTES (*P* = 0.0104), MIP1β (*P* = 0.0207), and IL-17A (*P* = 0.0499) were significantly higher than that of the PCS vaccine and/or the Gag/Env vaccine groups (Figures 6–9), while only the level of RANTES in the PCS vaccine group was higher than that of the Gag/Env vaccine group (*P*=0.0104) (Figure 8G). The increase of CVL cytokine/chemokine levels after prime did not significantly alter the relative differences among groups (time points showing non-significant differences were omitted in figures). Similarly, the significant increase in cytokine/chemokine levels in the Gag/Env vaccine group following Boost 1 did not result in alteration of relative levels of cytokines/ chemokines among the three vaccine groups.

After boosting with nanoformulated immunogens, the relative levels of CVL cytokine/chemokine levels between vaccine groups were significantly altered. The increase of IP-10 after NANOgag/env intranasal boost resulted in significant higher levels in comparison to the PCS vaccine group at week 17 (*P* = 0.0207) (Figure 6F). Similarly, significantly higher levels of IL-1β were observed in the Gag/Env vaccine group when compared to that of the PCS group at the same time point (*P* = 0.0379) (Figure 7D). Thus, boosting with nanoformulated Gag/Env DNA increased cervico-vaginal mucosal IP-10 and IL-1β levels.

On the other hand, the increase in MCP-1 following intranasal NANOpcs boost resulted in significantly higher levels of MCP-1 in the PCS vaccine group than that in the Gag/Env group (*P* = 0.0471) at week 51 (Figure 7J). In the case of IL-8, an increase in the levels of the Gag/Env group is observed over time (Figures 6I–L), reaching significant differences with the PCS group at week 52 (Figure 6J). Boost 3 also enhanced the relative difference in IL-1β between the Gag/Env and PCS groups (*P* = 0.0379) (Figure 7E). The repeated boosts also altered relative levels of MIP-1α (Figures 8A–F), and RANTES (Figures 8G–L) among the three groups.

## Discussion

Using a non-human primate model, our study is the first to demonstrate distinct SIV immunogens induced different mucosal inflammatory responses. SIV immunogens delivered with VSV and nanoformulation differentially influenced cervico-vaginal inflammatory cytokine/chemokine levels. MCMs immunized with full Gag and Env immunogens showed increase of a broader spectrum of inflammatory mediators with higher magnitude than those immunized with short PCS peptides. This elevation of multiple pro-inflammatory cytokines/chemokines in the cervico-vaginal mucosa of the Gag/Env-immunized MCMs persisted even at 18 weeks after the final boost; while repeated immunizations with the short PCS peptides had little persistent effect on the cervico-vaginal mucosal inflammatory mediators. The persistent elevation of pro-inflammatory mediators may have deleterious effect on potential efficacy conferred by the Gag/Env vaccine. The immunogen-induced mucosal inflammatory environment may tip the balance between vaccine-induced anti-viral effect and vaccine-induced susceptibility due to immune activation and the increase of target cells, thus influence vaccine efficacy. The findings in this study demonstrated that a candidate HIV vaccine may need to consider immunogen induced mucosal inflammation and immune activation.

Our study showed that both the VSV vector and the immunogens appear to contribute to the magnitude and spectrum of the increase of mucosal inflammatory mediators. Although multiple cytokines/chemokines slightly increased in the VSV vector-only control group after prime, only the level of IL-6 was increased >3-fold, whereas, >3-fold increase of five cytokines/chemokines (IFNγ, RANTES, GM-CSF, MCP-1, and IL-17A) was observed in the MCM immunized with rVSVpcs. The modest effect of rVSVgag/env on the mucosal inflammatory cytokines/chemokines after prime could be due to the slower replication of rVSVgag and rVSVenv than the VSV and rVSVpcs. The unique characteristic of VSV as vaccine vector to induce strong immunogen-directed humoral and cellular immune responses (20, 28, 29) largely depends on its fast replication (30). However, it has been demonstrated that although flexible packaging capacity is a major advantage in using VSV as a vaccine vector, adding inserts often led to slower viral replication and therefore, low viral titres (29). It is possible that due to carrying larger inserts, the rVSVgag/env initially induced a weaker and delayed innate immune response after prime, as seen in a previous study comparing cytotoxic T lymphocytes (CTL) responses between VSV-Gag/Env and VSV-Gag only immunization (20).

Our study also revealed that the influence of VSV vector on mucosal inflammatory mediators was short-lived, as the increase of cytokines/chemokines after the prime did not last, and further boost with the VSV vector had little effect. On the other hand, the apparent influence of immunogens on mucosal inflammatory mediators seemed to last much longer as demonstrated by the persistent elevation of multiple cytokines/chemokines 6–8 months after boosting with the Gag/Env vaccine. Although, when nano-formulated immunogens were used alone or together with VSV-vectored immunogens to boost the immune responses, the changes of the cervico-vaginal inflammatory cytokines/chemokines are likely due to the host response to the immunogens. Previous studies have shown that IgGs to VSV were rapidly induced after immunization with VSV vector and VSV-vectored immunogens, and neutralizing antibodies against VSV are detectable even at 300 days post-immunization (28, 31). Thus, the short-lived effect of VSV vector on the mucosal inflammatory mediators could be due to the induced anti-VSV immune responses. However, the anti-VSV vector immune responses did not appear to affect the influence of immunogens on the cervico-vaginal inflammatory cytokines/chemokines. Over the course of prime and boosts, the PCS and Gag/Env vaccines differentially influenced cervico-vaginal mucosal inflammatory cytokines/chemokines. Because only VSV-vectored immunogens were used for prime and Boost 4, and only nano-formulated immunogens were used for Boost 2, the influence of vector and immunogens can be inferred.

Mucosal inflammation is not typically included in vaccine efficacy evaluation. This study shows that evaluating the potential effects of vaccine immunogens on mucosal inflammatory cytokine/chemokine levels is important and that the balance between vaccine-induced anti-viral immune responses and vaccine-induced inflammatory response contributing to susceptibility needs to be considered in HIV vaccine development. However, since this study only examined the levels of cytokines/chemokines produced after immunization and boosting, this study was not able to determine which immune cells are producing specific cytokines/chemokine at a given time point. Future studies may examine mucosal tissue for cell activation and proliferation, as well as the evaluation of systemic inflammation following immunization.

## Data Availability Statement

All datasets presented in this study are included in the article.

## Ethics Statement

The non-human primate experiments were approved by the University of Wisconsin IACUC protocol (G005765) in accordance with the US Animal Welfare Act and following the recommendations of the National Research Council Guide for the Care and Use of Laboratory Animals, 8th Edition and the Weatherall report, The Use of Non-human Primates in Research. The Wisconsin National Primate Research Center is fully accredited by AAALAC under the University of Wisconsin, Division of Vice-Chancellor for Research and Graduate Education.

## Author Contributions

ML designed and coordinated the study, and reviewed the manuscript with NS-D, MA, QL, JW, DS, and JC-C. NT performed the experiments, interpreted the data, and wrote the manuscript. HL prepared rVSVs and with RO and MK reviewed the manuscript. NS-D coordinated animal study with DS. MA, JC-C, and TD provided nanoformulations. ML, FP, NS-D, MA, and JW secured funding for this study. All authors contributed to the article and approved the submitted version.

## Conflict of Interest

The authors declare that the research was conducted in the absence of any commercial or financial relationships that could be construed as a potential conflict of interest.
